# Assessing the needs of informal caregivers of patients with chronic non‐communicable diseases: A systematic review of self‐assessment tools

**DOI:** 10.1002/nop2.2008

**Published:** 2023-10-03

**Authors:** Panpan Yang, Mengzhen Ma, Qingyi Guan, Xingbin Du, Yanyan Fan

**Affiliations:** ^1^ School of Nursing Binzhou Medical University Yantai Shandong China

**Keywords:** chronic non‐communicable diseases, informal caregivers, needs assessment, psychometric properties, systematic review, tool

## Abstract

**Aim:**

To synthesize self‐administrated needs assessment tools of informal caregivers for patients with chronic non‐communicable diseases (CNCDs), evaluate the quality of psychometric properties and identify main needs assessment themes.

**Design:**

Systematic review.

**Methods:**

Eight electronic databases both in English and Chinese were searched for. The psychometric properties of tools were evaluated according to the quality criteria for good psychometric properties developed by Terwee et al. Both the content analysis and thematic extraction methods were used. Needs assessment themes were categorized based on the 7‐level Maslow's Hierarchy of Needs Theory.

**Results:**

A total of 17 tools were synthesized. Thirteen of them targeted informal caregivers of patients with cancer. The psychometric properties evaluated for most of these tools were content validity, internal consistency and construct validity. A total of 27 needs themes were identified and matched to six levels based on the 7‐level Maslow's Hierarchy of Needs theory, besides the aesthetic needs level.

**No Patient or Public Contribution:**

No primary data are being collected.

## 1 INTRODUCTION

Chronic non‐communicable diseases (CNCDs) such as cancer, diabetes, cardiovascular disorders and lung disease are expensive to treat, and often require lifelong care (Nugent, 2019). CNCDs are recognized as the greatest health care challenge of the 21st century (Tulu et al., 2021). According to the data from the household economic expenditure of CNCDs in 18 countries, per capita health expenditures exceed 40% of per capita effective income (Murphy et al., 2020). In addition, overall US costs of chronic disease are projected to accumulate by 2030 to more than $42 trillion, with medical outlays and productivity losses costing $8600 per person (‘What is the Impact of Chronic Disease on America’?). Furthermore, as many as 95% of patients with CNCDs live in their homes, and they require an informal caregiver for support with daily activities and symptom management due to the complexity of the disease and the low level of self‐care ability (Sarabia‐Cobo et al., 2020).

Informal caregivers are individuals who provide unpaid care and support for patients with medical, behavioural, disability or other condition(s) (Mollica et al., 2020). They provide a valued service to family members and society (Lefranc et al., 2017). For instance, in regard to their economic contribution, the estimated value of informal caregiving exceeds public expenditure on formal care and care allowances (Lefranc et al., 2017). However, studies have shown that informal caregivers also exerted great health costs (Lefranc et al., 2017; Petrovic & Gaggioli, 2020). Therefore, in 2018 ‘Research Priorities in Caregiving Summit’ convened by the Family Caregiving Institute at the Betty Irene Moore School of Nursing at UC Davis (University of California, Davis) (1) called for increasing awareness of informal caregivers and conducting needs assessment of the changing needs of informal caregivers over the trajectory of caregiving for tailored support for them (Family Caregiving Institute, 2018).

Needs assessment allows individuals to indicate the extent to which their needs across different areas have or have not been met, allowing for estimations of the prevalence of needs and the extent to which help is required. And the self‐assessment tools especially have the following advantages: not requiring in‐person contact with a trained clinician, better reflecting what one's real thoughts, and requiring less time, expense and space than the objective‐assessment (Newton et al., 2002).

Studies showed that healthcare staff support, information support, psychological support and communication skills improvement were the urgent but unmet needs (Akgun‐Citak et al., 2020; Hodson et al., 2019; Lee & Lee, 2020; Sarabia‐Cobo et al., 2020; Wang et al., 2018), which negatively affected caregivers' life and led to burden in the physiological, psychological and social levels (Akgun‐Citak et al., 2020; Zarit et al., 1986), such as unable to maintain optimal physical and mental well‐being (57%–78%), lost freedom (72%–75%) and work interruption (25%) (Jackson et al., 2018; Lefranc et al., 2017). Therefore, enhancing needs assessment and strategies development to support and train informal caregivers are necessary (Cianfrocca et al., 2018).

## 2 BACKGROUND

However, there was little synthesized information about how many instruments were developed for informal caregivers' needs (Lefranc et al., 2017), and what the most frequent needs themes assessed. Thus, a systematic review of the existing needs assessment tools of informal caregivers might be necessary to demonstrate overview of caregiver's needs. To date, there are some reviews (Lefranc et al., 2017; Mansfield et al., 2017; Prue et al., 2015) have reviewed studies of assessment tools for caregiver's needs and were published in 2015 and 2017 respectively. However, they only included English studies published until 2013 or 2016. Two of them (Mansfield et al., 2017; Prue et al., 2015) limited the target population to informal caregivers of people with dementia or cancer, thus they did not provide a bird's eye view of the informal caregiver's needs of patients with CNCDs. Another literature review (Lefranc et al., 2017) regarding eight self‐administered needs assessment tools of informal caregivers, based on their title and content explanations that were provided in eight articles, and identified the seven most frequent categories of caregiver needs: Health and Care, Psychological‐Emotional support, Information‐Knowledge, Social Life‐Work‐Finance, Future‐Bereavement‐Spirituality, Instrumental Support‐Respite and Satisfaction. But, this literature review only searched Medline database on September 2016. Therefore, the use of additional databases was suggested to find other self‐administered tools (Lefranc et al., 2017).

Furthermore, standardized tools should be valid, reliable and acceptable to respondents (Keszei et al., 2010). Thus, to ensure that tools assessing the informal caregiver's needs of patients with CNCDs produce data that is accurate, and comprehensive, their psychometric properties should be investigated and evaluated.

Therefore, the aim of the review was to synthesize self‐administrated needs assessment tools for informal caregivers of patients with CNCDs, evaluate their psychometric properties and extract the main needs assessment themes based on the comprehensive and systematic search of Chinese and English databases.

### 2.1 Research question

Specially, our research questions include:

- How many self‐assessment tools are developed for assessing informal caregivers' needs with CNCDs patients at present? And what are the characteristics of them (e.g. country, the number of items and domains, conceptual model and completion time)?
- How are the psychometric properties of these needs self‐assessment tools?
- What need themes do these self‐assessment tools incorporate?

## 3 MATERIALS AND METHODS

### 3.1 Design

A systematic review.

### 3.2 Method

This review was guided by Preferred Reporting Items for Systematic Reviews and Meta‐Analyses (PRISMA) guidelines (See File S1) and was registered with PROSPERO (CRD42022296584; https://www.crd.york.ac.uk/prospero/).

#### 3.2.1 Search strategies

Literature search was conducted in four English literature databases (PubMed, Cumulative Index to Nursing and Allied Health Literature [CINAHL], EMBase, Web of Science), four Chinese literature databases (Wan Fang Data, China National Knowledge Infrastructure [CNKI], Chongqing VIP [CQVIP], Chinese Biomedical Literature Database [CBM]) and Open Grey. All the articles from inception to August 2021 were considered. Before formal retrieval, multiple pre searches were carried out in each database to determine the retrieval strategy. The used MeSH terms and keywords included Non communicable diseases, noncommunicable disease, caregivers, spouses, family caregivers, family members, needs assessment, unmet needs, comprehensive needs, professional needs, care needs, scale, tool, instrument and the forth. The Boolean operator ‘OR’ and ‘AND’ were used to distinguish synonyms and combine search terms. The full search strategy of this systematic review is listed in Appendix S1.

#### 3.2.2 Selection process

All identified citations were collated and uploaded into NoteExpress database and duplicates were removed. Then, titles, abstracts and full text were screened and assessed against the eligibility criteria for the review by two independent reviewers (YPP, MMZ). Any disagreements that arose between the reviewers at each stage of the study selection process were resolved through discussion or with a third reviewer (FYY). The results of the search were presented in a Preferred Reporting Items for Systematic Reviews and Meta‐analyses (PRISMA) flow diagram (Moher et al., 2009).

#### 3.2.3 Eligibility criteria

(1) Study type: Original studies for the development of the needs self‐assessment tool and published in peer‐reviewed journals or in format of grey literature; (2) Population: Informal caregivers of patients with CNCDs; (3) Language: English or Chinese; (4) Outcome: Refer to the content and psychometric properties of the tool; (5) Accessible full texts.

#### 3.2.4 Data extraction

Two reviewers (YPP, GQY) independently completed data extraction by using a data extraction sheet. Data extraction mainly included: tool name, first author, year of publication, country of study, chronic diseases of focus, initial test population, conceptual model, the number of items and dimensions, time spent, assessment phases and psychometric properties. To ensure the comprehensiveness of needs themes, all included studies, regardless of their quality, were included in the synthesis. Any disagreements that arose between the reviewers were resolved through discussion, or with a third reviewer (FYY).

#### 3.2.5 Quality appraisal

The COSMIN checklist was meant for evaluating the methodological quality of a study on the measurement properties of a tool, not for evaluating the quality of the tool itself (Mokkink et al., 2010). To assess the quality of the tool, criteria for what good psychometric properties should be were previously published by members (Terwee et al., 2007) of this group (Mokkink et al., 2010). Considering this review focused on the tool itself, not the methodological quality of a study, thus, the psychometric properties quality of included tools was appraised according to the criteria developed by Terwee et al. (2007). This criterion included nine psychometric properties, each of which was rated as positive, intermediate, negative and no information respectively. The exact definitions of the psychometric properties and scoring criteria can be found in Table 1. Any disagreements that arose between the reviewers (YPP, DXB) were resolved through discussion, or with a third reviewer (FYY).

**TABLE 1:** Quality criteria for psychometric properties of tools.

Property	Definition	Quality criteria
Content validity	The extent to which the domain of interest is comprehensively sampled by the items in the questionnaire	Positive: A clear description is provided of the measurement aim, the target population, the concepts that are being measured and the item selection AND target population and (investigators OR experts) were involved in item selection; Indeterminate: A clear description of above‐mentioned aspects is lacking OR only target population involved OR doubtful design or method; Negative: No target population involvement; No information found on target population involvement.
Internal consistency	The extent to which items in a (sub)scale are intercorrelated, thus measuring the same construct	Positive: Factor analyses performed on adequate sample size (7 * # items and > 100) AND Cronbach's alpha(s) calculated per dimension AND Cronbach's alpha(s) between 0.70 and 0.95; Indeterminate: No factor analysis OR doubtful design or method; Negative: Cronbach's alpha(s) <0.70 or >0.95, despite adequate design and method; No information found on internal consistency.
Construct validity	The extent to which scores on a particular questionnaire relate to other measures in a manner that is consistent with theoretically derived hypotheses concerning the concepts that are being measured	Positive: Specific hypotheses were formulated AND at least 75% of the results are in accordance with these hypotheses; Indeterminate: Doubtful design or method (e.g. no hypotheses); Negative: Less than 75% of hypotheses were confirmed, despite adequate design and methods; No information found on construct validity.
Criterion validity	The extent to which scores on a particular questionnaire relate to a gold standard	Positive: Convincing arguments that gold standard is “gold” AND correlation with gold standard >0.70; Indeterminate: No convincing arguments that gold standard is “gold” OR doubtful design or method; Negative: Correlation with gold standard <0.70, despite adequate design and method; No information found on criterion validity.
Reproducibility	Agreement: The extent to which the scores on repeated measures are close to each other (absolute measurement error)	Positive: MIC < SDC OR MIC outside the LOA OR convincing arguments that agreement is acceptable; Indeterminate: Doubtful design or method OR (MIC not defined AND no convincing arguments that agreement is acceptable); Negative: MIC > SDC OR MIC equals or inside LOA, despite adequate design and method; No information found on criterion validity.
	Reliability: The extent to which patients can be distinguished from each other, despite measurement errors (relative measurement error)	Positive: ICC or weighted Kappa >0.70; Indeterminate: Doubtful design or method (e.g. time interval not mentioned); Negative: ICC or weighted Kappa <0.70, despite adequate design and method; No information found on criterion validity.
Responsiveness	The ability of a questionnaire to detect clinically important changes over time	Positive: SDC or SDC < MIC OR MIC outside the LOA OR RR >1.96 OR AUC≥0.70; Indeterminate: Doubtful design or method; Negative: SDC or SDC > MIC OR MIC equals or inside LOA OR RR <1.96 OR AUC <0.70, despite adequate design and methods; No information found on responsiveness.
Floor and ceiling effects	The number of respondents who achieved the lowest or highest possible score	Positive: ≤15% of the respondents achieved the highest or lowest possible scores; Indeterminate: Doubtful design or method; Negative: > 15% of the respondents achieved the highest or lowest possible scores, despite adequate design and methods; No information found on interpretation.
Interpretability	The degree to which one can assign qualitative meaning to quantitative scores	Positive: Mean and SD scores presented of at least four relevant subgroups of patients and MIC defined; Indeterminate: Doubtful design or method OR less than four subgroups OR no MIC defined; No information found on interpretation.

*Note*: Doubtful design or method = lacking of a clear description of the design or methods of the study, sample size smaller than 50 subjects (should be at least 50 in every (subgroup) analysis), or any important methodological weakness in the design or execution of the study.

Abbreviations: LOA, limits of agreement; MIC, minimal important change; SDC, smallest detectable change; ICC, Intraclass correlation; SD, standard deviation.

#### 3.2.6 Thematic extraction and clustering

The needs themes were extracted based on the method of qualitative content analysis: classifying large amounts of text into an efficient number of categories that represent similar meanings (Hsieh & Shannon, 2005). The items and domains of each tool were marked. Items with similarity in meaning from all included tools were put into the same pool. Each pool was named with a need theme and then were matched according to the definition of each level of 7‐level Maslow's hierarchy of needs theory(Goble, 1970; Maslow, 1943): (1) Physiological needs are the most fundamental needs, namely, air, water, food, shelter and rest. (2) Safety needs include the contemporary issues of personal and financial security. (3) Friendly and intimate relationships with people in general and identifying with a particular group or groups are love and belonging needs. Love needs involve both giving and receiving love. (4) Esteem needs, a need for a stable, firmly based, high evaluation of themselves, based upon real capacity, achievement and respect from others. (5) Cognitive needs are a desire to understand, to systematize, to organize to analyse and to look for relations and meanings. (6) The need for aesthetics is related to people's self‐image, and it helps people become healthier. (7) Self‐actualization needs, the realization of one's full potential in realms such as athletics, poetry or science, et al, to realize personal meaning of life. Reviewers (YPP, MMZ, FYY) resolved discrepancies in needs extraction and clustering through regular discussions.

### 3.3 Ethics

Ethical approval is not required as no primary data are being collected.

## 4 RESULTS

**FIGURE 1:**
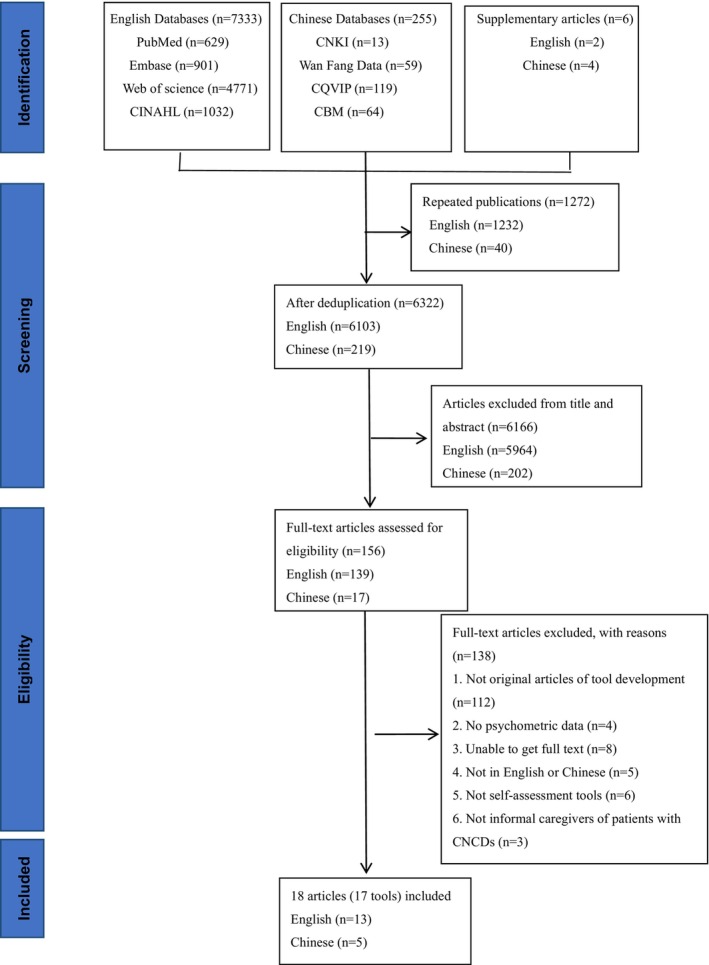
PRISMA flow diagram.

Our search yielded 7594 results. All citations were collated and uploaded into the NoteExpress database and the 1272 duplicates were removed. Then, titles and abstracts of articles identified in the search were screened and 6166 articles were excluded because of no related to the research topic. Of the remaining 156 articles, 138 articles were excluded after a full‐text evaluation according to the inclusion and exclusion criteria. Finally, a total of 18 articles with 17 needs assessment tools were included because the development of tool CaTCoN (Lund et al., 2012, 2014) was reported in two articles from different aspects. Figure 1 shows the process of querying the database and of article selection.

### 4.1 Quality appraisal results

The description of each tool’ psychometric properties is presented in Table 2. Table 3 presented tool quality appraisal results.

**TABLE 2:** Characteristics of psychometric properties.

Tool name	Content validity					Construct validity	Internal consistency	Criterion validity	Reproducibility	Floor and ceiling effects	Interpretability
	Measurement aim	Target population	Concepts	Item selection and item reduction							
				Population involved	Methods				Agreement		
Scale of Need (SON) (Yen, 2008)	To assess the areas where caregivers of mentally ill patients need support	Caregivers of mentally ill patients	Caregiver needs	1 psychiatric nurse; 1 psychiatrist; 2 psychiatric nurses; 1 caregiver of a schizophrenic patient; investigators	Literature review, Expert review	Exploratory factor analysis: accounted for 66.12% of the total variance	α = 0.92 Subscales: α range from 0.70 to 0.80	‐	‐	‐	‐
Supportive Care Needs Survey‐Partners and Caregivers (SCNS‐P&C) (Girgis et al., 2011)	To assess the multi‐dimensional supportive care needs of cancer caregivers	Cancer caregivers	‐	Psycho‐oncology experts; members of the general public; and caregivers of cancer survivors; investigators	Literature review, Existing Instruments, Expert review	Known‐group approach: 75% hypothesis was supported	Subscales: α range from 0.88 to 0.94	‐	‐	‐	‐
Needs Assessment of Family Caregivers‐Cancer (NAFC‐C) (Kim et al., 2010)	To assess family caregivers' needs across the different survivorship phases	Cancer family caregivers	‐	Principal investigator; 1666 caregivers	Existing tools; New items that the Principal Investigator identified	Exploratory factor analysis: accounted for 58.4% of the total variance	Subscales: α range from 0.56 to 0.86	‐	‐	‐	Reporting the prevalence of unmet needs and the influencing factors at 2‐month, 2‐year and 5‐year cohorts respectively
Comprehensive Needs Assessment tool for Cancer Caregivers (CNAT‐C) (Shin et al., 2011)	To assess comprehensive needs for caregivers of cancer patients	Family caregivers of cancer patients	‐	Oncology physicians; nurses; psychiatrists; psychologists; social workers involved in cancer patient care; family caregivers of cancer patients with different cancer types and treatment stages; investigators	Literature review, Panel of experts review, caregiver interview, pilot test	Principal factor analysis: accounted for 66.4% of the total variance	α = 0.96 Subscales: α range from 0.79 to 0.95	‐	‐	‐	Caregiver's needs scores (Mean ± SD) in different caregiver characteristics
Cancer Survivors' Partners Unmet Needs measure (CaSPUN) (Hodgkinson et al., 2007)	To assess the long‐term supportive care needs for cancer survivors' partners	Cancer survivors' partners	‐	3 health professionals; 2 two research academics; 1 consumer; 5 partners of cancer survivors; investigators	Literature review, qualitative study, panel of experts review	Examining hypothesized relationships between needs and partner variables: 80% hypothesis was supported	α = 0.94 Subscales: α range from 0.79 to 0.90	‐	Test–retest correlations range from 0.08 to 0.53	‐	The CaSPUN can be scored and interpreted in regard to items or domains of need, and in regard to the sum of met, unmet and total needs.
Cancer Support Person's Unmet Needs Survey (SPUNS) (Campbell et al. 2009)	To assess the unmet needs of those persons identified by a cancer survivor as the principal individual who is providing support to them during their survivorship period	Cancer survivors' principal support persons	‐	74 cancer survivors and their principal support persons; expert panel of epidemiologists, behavioural scientists, psychosocial professionals, and Canadian Cancer Society staff; investigators	Literature review, expert panel review, Pilot Test	Factor analysis: accounted for 73.5% of the total variance	α = 0.99 Subscales: α range from 0.93 to 0.98	‐	All items test–retest Correlations >0.70	‐	‐
Home Caregiver Need Survey (HCNS) (Hileman et al., 1992)	To assess the needs of caregivers of cancer patients at home	Family caregivers of cancer patients	Need, Information Needs et al	15 patients; 15 home caregivers; investigators	Panel of experts, Statement from patients and home caregivers	‐	α = 0.93 Subscales:α range from 0.85 to 0.97	‐	‐	‐	‐
Dementia Carer Assessment of Support Needs Tool (DeCANT) (Clemmensen et al., 2021)	To assess the support needs of carers of people with dementia	Carers of people with dementia	Carers' support needs	23 carers; 13 professionals; 3 researchers; The expert panel: representative of dementia carers in general, or professionals in the area of dementia from different professions and from different care settings; investigators	Literature review, qualitative study, Input from experts, professionals, carers of dementia patients	Confirmatory factor analysis: Model 1: CFI:0.934 TLI:0.927 WRMR:1.342 PRSEA:0.083 Model 2: CFI:0.932 TLI:0.925 WRMR:1.393 PRSEA:0.084 Post hoc analysis of Model 2: CFI:0.946 TLI:0.938 WRMR:1.265 PRSEA:0.073	Subscales: α range from 0.73 to 0.84	‐	‐	‐	‐
Cancer Caregiving Tasks, Consequences and Needs Questionnaire (CaTCoN) (Lund et al., 2012, 2014)	To assess the caregiving tasks and consequences, and the caregivers' needs	Caregivers of cancer patients	Needs, Caregivers	Cancer patients' caregivers; cancer patients; clinicians and cancer counsellors; investigators	Literature review, Focus group interviews, Cognitive interviews	The hypothesized convergent CaTCoN and FAMCARE/FIN subscales correlated 0.59–0.74; The hypothesized divergent CaTCoN and FAMCARE/FIN subscales correlated −0.11–0.25	Subscales:α range from 0.65 to 0.95	‐	‐	‐	‐
Head and Neck Information Needs Questionnaire (HaNiQ) (Dall'Armi et al., 2013)	To assess the needs of head and neck cancer patients and their caregivers	Neck cancer patients and their caregivers	‐	Investigators	Adapting the information needs questionnaire for breast cancer patients	‐	α = 0.94 Subscales: α range from 0.73 to 0.89	‐	‐	‐	‐
Caregiver Needs Screen (CNS) (Boele et al., 2019)	To assess the needs family caregivers of patients with a primary malignant brain tumour	Family caregivers of patients with a primary malignant brain tumour	‐	109 family caregivers of patients with a primary malignant brain tumour; experts in caregiving, neurosurgery, neuro‐oncology and methodology; investigators	Qualitative study, experts review	Principal component analysis: accounted for 75.8% of the total variance	Subscales: α range from 0.65 to 0.86	The correlations between the CNS and the CES‐D ranging from r = 0.43–0.67; The correlations between the CNS and the POMS‐A ranging from r = 0.41–0.63		With percentages of minimum and maximum scores ranging from 0.8% to 9.2%, only one domain found floor effect: 26.1% of participants had a minimum score	
Family Inventory of Needs (FIN) (Kristjanson et al., 1995)	To assess the importance of care needs of families of Advanced cancer patients and the extent to which families perceive that their care needs have been met	Family caregivers of advanced cancer patients	Importance of Family Care Needs, Fulfilments of Care Needs	6 experts (family members of advanced cancer patients); investigators	Expert panel review	‐	α = 0.83	‐	‐	‐	‐
Tool of supportive services needs for elderly family carers with chronic diseases in Yunnan province (Yan 2019b)	To assess senile chronic disease in Yunnan province family caregivers support services need	Family caregivers of elderly patients with chronic disease	Family caregivers, support services, needs	13 primary family caregivers of elderly patients with chronic disease; 5 experts (chief nurses); investigators	Literature review, Delphi method, Qualitative study, Pre experiment	Exploratory factor analysis: accounted for 77.01% of the total variance	α = 0.95 Subscales: α range from 0.88 to 0.99	‐	Test–retest Correlation = 0.801	‐	To analyse the needs of caregivers and their influencing factors
Tool of Professional Needs of Care for Family Caregivers of Stroke (Tianyue, 2019)	To explore the level and influencing factors of the caregiver's professional needs	Family Caregivers of patients with Stroke	Family Caregivers of patients with Stroke, professional care, Caregivers' professional needs	17 family caregivers of patients with stroke; 4 stroke research experts; 3 community nursing experts; 2 community nursing managers; 2 tool development experts; 2 clinical medical experts; 2 clinical nursing experts; investigators	Literature review, Delphi method, Qualitative study, Pre experiment	Exploratory factor analysis: accounted for 67.34% of the total variance	α = 0.92 Subscales: α range from 0.82 to 0.95	‐	Test–retest correlations range from 0.849 to 0.980	‐	To explore the level and influencing factors of caregiver's professional needs.
Supportive needs questionnaire for caregivers with malignant blood disease (Yan 2019a)	To assess blood tumour caregivers support needs	Blood tumour caregivers	Caregiver, Need, Supportive care needs	10 blood tumour caregivers; 3 Nursing education experts; 10 clinical nursing; 4 clinical medical experts; investigators	Literature review, Delphi method, qualitative study, Pre experiment	Exploratory factor analysis: accounted for 66.6% of the total variance	α = 0.90 Subscales: α range from 0.75 to 0.83	‐	Test–retest correlations range from 0.724 to 0.807	‐	To explore the level and influencing factors of caregiver's professional needs.
Needs assessment scale for spouses of female breast cancer patients (Yonghui et al., 2019)	To assess the needs of female breast cancer patients' spouses	Female breast cancer patients' spouses	Needs	10 female breast cancer patients' spouses; 5 doctors; 4 nursing educators; 6 nursing managers; investigators	Literature review, Delphi method, qualitative study, Pre experiment	Exploratory factor analysis: accounted for 68.32% of the total variance	α = 0.93 Subscales: α range from 0.68 to 0.93	‐		‐	
Care Needs Questionnaire of Main Caregivers of Postoperative Patients with Gastric Cancer (Junhua 2021)	To investigate the care needs of the main caregivers of patients with gastric cancer after operation	The main caregivers of patients with gastric cancer after operation	Needs; The main caregivers	15 main caregivers of patients with gastric cancer after operation; 2 clinical medical experts; 9 clinical nursing; 4 nursing managers; 5 nursing educators; investigators	Literature review, Delphi method, qualitative study， Pre experiment	Exploratory factor analysis: accounted for 71.243% of the total variance	α = 0.896 Subscales: α range from 0.60 to 0.90	‐	Test–retest correlations 0.879	‐	To explore the level and influencing factors of the care needs of the main caregivers

**TABLE 3:** The results of quality evaluation.

Tool name	Content validity	Internal consistency	Construct validity	Criterion validity	Reproducibility (agreement)	Floor and ceiling effects	Interpretability
SON (Yen, 2008)	+	+	−	0	0	0	0
SCNS‐P&C (Girgis et al., 2011)	?	+	+	0	0	0	0
NAFC‐C (Kim et al., 2010)	?	−	?	0	0	0	?
CNAT‐C (Shin et al., 2011)	?	+	−	0	0	0	?
CaSPUN (Hodgkinson et al., 2007)	?	+	+	0	−	0	?
SPUNS (Campbell et al., 2009)	+	−	−	0	+	0	0
HCNS (Hileman et al., 1992)	?	−	0	0	0	0	?
DeCANT (Clemmensen et al., 2021)	+	+	+	0	0	0	0
CaTCoN (Lund et al., 2012, 2014)	+	−	−	?	0	0	0
HaNiQ (Dall'Armi et al., 2013)	−	?	0	0	0	0	0
CNS (Boele et al., 2019)	?	−	+	?	0	+	0
FIN (Kristjanson et al., 1995)	+	?	?	0	0	0	0
Tool of supportive services needs for elderly family carers with chronic diseases in Yunnan province (Yan 2019b)	+	−	+	0	+	0	?
Tool of Professional Needs of Care for Family Caregivers of Stroke (Tianyue, 2019)	+	+	−	0	+	0	?
Supportive needs questionnaire for caregivers with malignant blood disease (Yan 2019a)	+	?	−	0	+	0	0
Needs assessment scale for spouses of female breast cancer patients (Yonghui et al., 2019)	+	−	−	0	0	0	0
Care Needs Questionnaire of Main Caregivers of Postoperative Patients with Gastric Cancer (Junhua 2021)	+	−	−	0	+	0	?

*Note*: “+”, positive rating; “?”, indeterminate rating; “−”, negative rating; “0”, no information available.

Content validity was documented in all included tools. According to the criterion of Terwee et al. (2007), 10 tools (59%) (Campbell et al., 2009; Clemmensen et al., 2021; Junhua, 2021; Kristjanson et al., 1995; Lund et al., 2012; Tianyue, 2019; Yan, 2019a, 2019b; Yen, 2008; Yonghui et al., 2019) were evaluated as positive because of a clear description of the measurement aim, the concepts that are being measured, the target population and experts team during the items selection process (Terwee et al., 2007); Six tools(35%) (Boele et al., 2019; Campbell et al., 2009; Girgis et al., 2011; Hodgkinson et al., 2007; Kim et al., 2010; Shin et al., 2011) were evaluated an indeterminate rating because of failing to provide the concept to be measured; One tool (Dall'Armi et al., 2013) was evaluated a negative rating because of not include the target population during the items selection process.

Cronbach's alpha of was calculated as an indicator of internal consistency. It was reported in all included tools. According to the criterion (Terwee et al., 2007), the condition that when factor analysis was applied, Cronbach's alpha per domain was reported, and its value was between 0.70 and 0.95 were necessary for the positive rating. Therefore, six tools (35%) (Clemmensen et al., 2021; Girgis et al., 2011; Hodgkinson et al., 2007; Shin et al., 2011; Tianyue, 2019; Yen, 2008) were rating positive; eight were rating negative (Boele et al., 2019; Campbell et al., 2009; Hileman et al., 1992; Junhua, 2021; Kim et al., 2010; Lund et al., 2014; Yan, 2019b; Yonghui et al., 2019), and three (Dall'Armi et al., 2013; Kristjanson et al., 1995; Yan, 2019a) were rating indeterminate.

Construct validity was assessed in 14 of the included tools according to the available information. Four tools (24%) (Boele et al., 2019; Girgis et al., 2011; Hodgkinson et al., 2007; Yan, 2019b) were given a positive rating, which formulated specific hypotheses and at least 75% of the results were in accordance with them. Eight tools had a negative rating (Campbell et al., 2009; Junhua, 2021; Kim et al., 2010; Shin et al., 2011; Tianyue, 2019; Yan, 2019a; Yen, 2008; Yonghui et al., 2019).

Reproducibility was evaluated for six tools (Campbell et al., 2009; Hodgkinson et al., 2007; Junhua, 2021; Tianyue, 2019; Yan, 2019a, 2019b) according to test–retest agreement, five of them (Campbell et al., 2009; Junhua, 2021; Tianyue, 2019; Yan, 2019a, 2019b) were evaluated a positive rating, with the test–retest correlations above 0.70. Criterion validity and floor and ceiling effects were only reported for one article (Boele et al., 2019), and were evaluated as an indeterminate rating and a positive rating, respectively, because of no convincing arguments that the gold standard is ‘gold’ and the minimum or maximum scores ranging from 0.8% to 9.2% in most domains. Six articles (Hileman et al., 1992; Hodgkinson et al., 2007; Junhua, 2021; Shin et al., 2011; Tianyue, 2019; Yan, 2019b) reported the interpretability and were given an indeterminate rating, because of lacking of the mean and standard deviation (SD) scores of relevant subgroups or a minimal important change (MIC) or less than four subgroups. Neither reliability in reproducibility or responsiveness was reported in the included studies.

### 4.2 Characteristics of the tools

Seventeen tools from six countries were identified. They were published in Chinese or English from 1989 to 2021, covering four specific CNCDs types. All tools relied on Likert scale‐type response devices. The completion time of the tools range from 5 to 30 min. Detailed description of tools, such as the authors, countries, initial test population and completion time is provided in Table 4.

1. Countries

**TABLE 4:** Characteristics of needs assessment tools.

Tool Name	Reference	Country	Care patient’ disease	Initial Test Population	Description	Conceptual model	Completion time
SON	Yen et al (2008)	China‐Taiwn	Mental illness	250 caregivers of mentally ill patients	23 items Measures needs in terms of one dimension ‐ 4 point Likert scale; Four domains: information need, supportive need, need for respite care, need for emotional release	_	Duration:<10 min
SCNS‐P&C	Girgis et al (2001)	Australia	Cancer	547 caregivers of patients with various cancers 6–8 months post‐diagnosis	40 items Measures needs in terms of one dimension ‐ 5 point Likert scale; Four domains:health care service needs, psychological and emotional needs, work and social needs, and information needs	_	_
NAFC‐C	Kim et al. (2010)	United States	Cancer	1666 caregivers of patients of the top 10 cancers divided into 3 cohorts: 2 months(*n* = 162), 2 years (*n* = 896) and 5 years (*n* = 608) post‐diagnosis	27 items Measures needs in terms of two dimensions ‐ 5 point Likert scale:importance of the need; how the need has been fulfilled；Four domains: psychosocial unmet needs, medical unmet needs, financial unmet needs and daily activity unmet needs	Need Fulfillment Theory	Duration:<10 min
CNAT‐C	Shin et al. (2011)	Korea	Cancer	600 family caregivers of patients with cancer during or after cancer treatment	41 items Measures needs in terms of one dimension ‐ 4 point Likert scale; Seven domains: health and psychological problems, family/social support, health‐care staff, information, religious/ spiritual support，practical support and hospital facilities and services	_	_
CaSPUN	Hodgkinson et al. (2007)	Australia	Cancer	212 spouses of individuals with various cancers 1–11 years post‐diagnosis	35 items Measures needs in terms of one dimension ‐ 5 point Likert scale; Five domains: relationships. Information, partner issues, comprehensive care and emotional support	—	10 min
SPUNS	Campbell et al. (2009)	Canada	Cancer	382 support persons of individuals with various cancers 12–60 months post‐diagnosis	78 items Measures needs in terms of one dimension ‐ 5 point Likert scale; Six domains:information and relationship needs, emotional needs, personal needs, work and finance, health care access and continuity and worries about future services	_	Duration:<15 min
HCNS	Hileman et al (1989)	United States	Cancer	492 family caregivers of individuals diagnosed with breast, colon, lung and oesophageal cancer 3 months to 9 years previously	90 items Measures needs in terms of two dimensions ‐ 7 point Likert scale:the importance and satisfaction of each need；Six domains:information, household, patient care, personal, spiritual, psychological	Lackey‐Wingate Model	30 min
DeCANT	Clemmensen et al. (2021)	Denmark	Dementia	301 informal carers of individuals diagnosed with dementia throughout the disease trajectory and across settings	25 items Measures needs in terms of one dimension ‐ 4 point Likert scale; Four domains: communicating and interacting with surroundings , daily life when caring for a person with dementia, maintaining own well‐being and focusing on themselves (MODEL 1) Environmental factors, activity and participation components, personal factors and body structure/function components (MODEL2)	Biopsychosocial Model	Duration:<10 min
CaTCoN	Lund et al. (2012)	Denmark	Cancer	590 caregivers of individuals with various cancers 6 months to more than 5 years	41 items Measures needs in terms of one dimension ‐ 4 point Likert scale; Nine domains: caregiving workload, lack of attention from HCPs on the caregivers wellbeing, lack of personal growth, lack of privacy during conversations with HCPs, need for help HCPs, problems with the quality of information and communication from HCPs, lack of information from HCPs, Need for contact to other caregivers, negative consequences of being a caregiver	Lazaros and Folkman stress‐coping theory	_
HaNiQ	Dall'Armi et al. (2013)	Australia	Head and Neck Cancer	52 caregivers of patients with head and neck cancer	33 items Measures needs in terms of one dimension ‐ 4 point Likert scale; Five domains: disease profile, treatment, side effects, psychosocial and survivorship	–	–
CNS	Boele et al. (2019)	United States	Neuro‐Oncology	122 caregivers of patients with primary malignant brain tumor	32 items Measures needs in terms of one dimension ‐ 10 point Likert scale; Six domains: neurological symptoms, oncologic symptoms, personal communication(friends,family), communicating with health care providers, resources and caregiver health	–	5–7 min
FIN	Kristjanson et al. (1995)	Canada	Advanced Cancer	109 family members of advanced cancer patients in hospice programs	20 items Measures needs in terms of two dimension:the importance of the need is rated along a 10 point Likert scale; the satisfaction of the need is rated along a dichotomous fulfillment scale (met/unmet); Two domains:importance of need subscale, need fulfillment subscale	Fulfillment Theory	–
Tool of supportive services needs for elderly family carers with chronic diseases in yunnan province	Li et al (2019)	China	Elderly Chronic Disease	205 family caregivers of elderly chronic disease	25 items Measures needs in terms of one dimension ‐ 5 point Likert scale; Four domains: professional medical support services needs, community support services needs, personal feelings needs, policy requirements needs	Lazaros and Folkman stress‐coping theory	3‐11 min
Tool of Professional Needs of Care for Family Caregivers of Stroke	Zhang et al (2020)	China	Stroke	571 family caregivers of patients with stoke	29 items Measures needs in terms of one dimension ‐ 5 point Likert scale; Four domains: stoke professional knowledge needs, complications nursing needs, psychological support needs, cognitive exercise needs	Bowen family systems theory; The Family Support Model	9 min
Supportive needs questionnaire for caregivers with malignant blood diseases	Guo et al (2019)	China	Malignant Blood	193 caregivers of patients with malignant blood diseases	29 items Measures needs in terms of one dimension ‐ 5 point Likert scale; Five domains: disease knowledge needs, caring skills, psychological needs, economy and medical treatment insurance needs, social support needs	–	–
Needs assessment scale for spouses of female breast cancer patients	Wan et al (2020)	China	Breast Cancer	169 spouses of patients with breast cancer diseases	23 items Measures needs in terms of one dimension ‐ 5 point Likert scale; Five domains: physiological needs, safety needs, belongingness and love needs, esteem needs and self‐actualization needs	Maslow's hierarchy of needs theory	–
Care needs questionnaire of main caregivers of postoperative patients with gastric cancer	Junhua (2021)	China	Gastric Cancer	120 main caregivers of postoperative patients with gastric cancer	18 items Measures needs in terms of one dimension ‐ 5 point Likert scale; Five domains: disease‐related knowledge education needs, activity guidance needs, multi‐support needs, diet guidance needs, psychological care needs	Maslow's hierarchy of needs theory	–

Six tools were developed in China (5 in mainland China; 1 in Taiwan) (Junhua, 2021; Tianyue, 2019; Yan, 2019a, 2019b, 2008; Yonghui et al., 2019), followed by three from the United States (Boele et al., 2019; Hileman et al., 1992; Kim et al., 2010) and Australia (Dall'Armi et al., 2013; Girgis et al., 2011; Hodgkinson et al., 2007), respectively, two in Denmark (Clemmensen et al., 2021; Lund et al., 2014) and Canada (Campbell et al., 2009; Kristjanson et al., 1995), respectively, and one in Korea (Shin et al., 2011) .

2 Languages

Five articles were published in Chinese (Junhua, 2021; Tianyue, 2019; Yan, 2019a, 2019b; Yonghui et al., 2019), and the rest were published in English.

3 Types of CNCDs

The types of CNCDs covered cancer (*n* = 13) (Boele et al., 2019; Campbell et al., 2009; Dall'Armi et al., 2013; Girgis et al., 2011; Hileman et al., 1992; Hodgkinson et al., 2007; Junhua, 2021; Kim et al., 2010; Kristjanson et al., 1995; Lund et al., 2014; Shin et al., 2011; Yan, 2019a; Yonghui et al., 2019), stroke (*n* = 1) (Tianyue, 2019), dementia (*n* = 1) (Clemmensen et al., 2021), mental illness (*n* = 1) (Yen, 2008) and chronic disease (*n* = 1) (no information on the specific type of chronic disease) (Yan, 2019b).

4 Needs level

Conceptual models show the logic, relationships among concepts (Casanave & Li, 2015), and using it can ensure the comprehensiveness and scientificity of the scale content. Generally, conceptual and theoretical models are used interchangeably (Casanave & Li, 2015). The development of nine tools in this review was based on conceptual models (Clemmensen et al., 2021; Hileman et al., 1992; Junhua, 2021; Kim et al., 2010; Kristjanson et al., 1995; Lund et al., 2012; Tianyue, 2019; Yan, 2019b; Yonghui et al., 2019), such as Need Fulfilment Theory, Lackey‐Wingate Model, Biopsychosocial Model, Lazaros and Folkman stress‐coping theory, Bowen family systems theory, The Family Support, and Maslow's hierarchy of needs theory. The assessed needs included: comprehensive needs (*n* = 11) (Boele et al., 2019; Campbell et al., 2009; Hileman et al., 1992; Hodgkinson et al., 2007; Junhua, 2021; Kim et al., 2010; Kristjanson et al., 1995; Lund et al., 2014; Shin et al., 2011; Yen, 2008; Yonghui et al., 2019), supporting needs (*n* = 4) (Clemmensen et al., 2021; Girgis et al., 2011; Yan, 2019a, 2019b), information needs (*n* = 1) (Dall'Armi et al., 2013) and professional care needs (*n* = 1) (Tianyue, 2019). The 17 multidimensional tools had an average of 5 ± 2 dimensions (min =2, max = 9), and 36 ± 20 items (min =18, max = 90).

5 Time spent and assessment phases

Nine tools gave the time spent (Boele et al., 2019; Campbell et al., 2009; Clemmensen et al., 2021; Hileman et al., 1992; Hodgkinson et al., 2007; Kim et al., 2010; Tianyue, 2019; Yan, 2019b; Yen, 2008). 37.2% of them took no more than 10 mins (Boele et al., 2019; Clemmensen et al., 2021; Hodgkinson et al., 2007; Kim et al., 2010; Tianyue, 2019; Yen, 2008), the other tools took from 5 to 7 mins (Boele et al., 2019) to 30 mins (Hileman et al., 1992). Five tools (Hileman et al., 1992; Kristjanson et al., 1995; Shin et al., 2011; Tianyue, 2019; Yan, 2019b) were designed to use in the setting of home. Four tools indicated specific needs assessment stages, such as at least 6 months post‐diagnosis (Lund et al., 2014), within 6–8 months (Girgis et al., 2011), 12–60 months post‐diagnosis (Campbell et al., 2009), at least 1 year post‐diagnosis (Hodgkinson et al., 2007).

### 4.3 Thematic extraction and clustering results

A total of 27 themes of needs were assessed by the tools (see Table 5). The number of needs themes covered by the needs assessment tools ranged from 1 to 14, of which DeCANT (Clemmensen et al., 2021) contributed the most to themes extraction. The most popular four needs themes were psychological‐emotional, information, communication and healthcare professionals support, which were concerned by 14 tools (Campbell et al., 2009; Clemmensen et al., 2021; Girgis et al., 2011; Hileman et al., 1992; Hodgkinson et al., 2007; Junhua, 2021; Kim et al., 2010; Lund et al., 2012, 2014; Shin et al., 2011; Tianyue, 2019; Yan, 2019a, 2019b; Yen, 2008; Yonghui et al., 2019), 14 tools (Campbell et al., 2009; Dall'Armi et al., 2013; Girgis et al., 2011; Hileman et al., 1992; Hodgkinson et al., 2007; Junhua, 2021; Kim et al., 2010; Kristjanson et al., 1995; Lund et al., 2012, 2014; Shin et al., 2011; Yan, 2019a, 2019b; Yen, 2008; Yonghui et al., 2019), nine tools (Boele et al., 2019; Clemmensen et al., 2021; Girgis et al., 2011; Hileman et al., 1992; Hodgkinson et al., 2007; Kim et al., 2010; Lund et al., 2012, 2014; Yan, 2019b; Yonghui et al., 2019), nine tools(Clemmensen et al., 2021; Girgis et al., 2011; Hodgkinson et al., 2007; Junhua, 2021; Kim et al., 2010; Lund et al., 2012, 2014; Shin et al., 2011; Yen, 2008; Yonghui et al., 2019) respectively. Medical insurance (Yan, 2019a) and value recognition (Yan, 2019b) only appeared in one assessment tool respectively.

**TABLE 5:** Themes distribution of needs assessment tools.

Categories of needs	Needs themes	Author/year of the tool																	Numbers of tools in each theme
		Yen (2008)	Girgis et al. (2011)	Kim et al. (2010)	Shin et al. (2011)	Hodgkinson et al. (2007)	Campbell et al. (2009)	Hileman et al. (1992)	Clemmensen et al. (2021)	Lund et al. (2012, 2014)	Dall'Armi et al. (2013)	Boele et al. (2019)	Kristjanson et al. (1995)	Yan (2019a)	Tianyue (2019)	G Yan (2019a)	Yonghui et al. (2019)	Junhua (2021)	
Physiological needs	Health				√	√			√			√	√			√	√		7
	Sleeping						√	√	√										3
	Sex		√			√			√										3
	Rest			√				√	√	√									4
Safety needs	Caregivers' finances			√		√	√		√	√		√				√		√	8
	Medical insurance															√			1
Love and belonging needs	Family support	√	√		√			√	√					√					6
	Communication		√	√		√		√	√	√		√		√			√		9
	Psychological ‐emotional	√	√	√	√	√	√	√	√	√				√	√	√	√	√	14
	Spiritual support				√	√		√				√				√			5
	Relationship			√		√	√	√	√							√	√		7
Esteem needs	Being involved in medical decisions		√	√	√														3
	Being respected				√								√						2
Cognition needs	Knowledge													√	√			√	3
	Information	√	√	√	√	√	√	√		√	√		√	√		√	√	√	14
	Caring skill							√		√					√	√			4
	Symptom management			√					√			√	√						4
Self‐actualization needs	Balancing roles			√		√	√		√										4
	Care‐value actualization					√											√		2
	Value recognition													√					1
	Hospital facilities and services	√			√		√		√	√				√					6
	Healthcare professionals support	√	√	√	√	√			√	√							√	√	9
	Social support	√			√			√								√			4
	Community support													√					1
	Work		√			√	√					√				√			5
	Legislation support	√							√					√					3
	Practical support				√	√		√											3
Numbers of themes in each tool		7	8	10	11	13	8	11	14	8	1	6	4	9	3	10	7	5	

*Note*: “√” means the theme is included in the tool.

Based on Maslow's hierarchy of needs theory, the 27 needs themes were mapped to six levels (see Table 5): physiological needs (4 themes), safety needs ((2 themes), love and belonging needs (5 themes), esteem needs (2 themes), cognition needs (4 themes) and self‐actualization needs (10 themes). Aesthetic needs were not reported and concerned in the included tools.

## 5 DISCUSSION

### 5.1 Characteristics of the tools

#### 5.1.1 Target population of assessment tools

This review demonstrated that there had been a growth in tool development targeting ‘caregivers' needs’ since 2010. Of note, these tools were more often focused on informal caregivers for patients with cancer, stroke and dementia. Nevertheless, 43% of overall deaths and 49% of adult deaths were estimated, by WHO, to be due to cardiovascular, respiratory or related disorder (Prabhakaran et al., 2018). Additionally, researches showed that caring for patients with cardiovascular or respiratory diseases had a significant burden on caregivers' lives, whose needs were often overshadowed and not being met (Jackson et al., 2018; Petrovic & Gaggioli, 2020). Therefore, the development of needs assessment tools targeted to other subpopulation of informal caregivers is necessary.

#### 5.1.2 Assessment phases

‘Timing It Right Theory’ pointed out the changing needs of caregivers in different care environments (Cameron et al., 2013). In this review, nine tools had been designed for use in the specific or environmental phase. For example, five tools (Hileman et al., 1992; Kristjanson et al., 1995; Shin et al., 2011; Tianyue, 2019; Yan, 2019b) were designed to use in the setting of home, which tailored to assess family caregivers' needs. The other four tools reported the assessment time based on the phases of patients' disease which ranged from 6 months to 60 months post‐diagnosis (Campbell et al., 2009; Girgis et al., 2011; Hodgkinson et al., 2007; Junhua, 2021; Lund et al., 2014). According to the Swore Fletcher model, it highlights the disease trajectory as an important element, and the stress is likely to be experienced differently in different phases (Fletcher et al., 2012). Therefore, the definition of the specific assessment phase based on the trajectory of different chronic diseases of patients is necessary in the tool development of informal caregivers' needs assessment.

### 5.2 Quality appraisal

In this review, the overall quality of the included 17 tools were not too satisfactory, it still needs to be improved. The advantages are that the common psychometric properties were all evaluated in all or most of the tools, such as content validity, internal consistency and construct validity on the one hand; on the other hand, content validity which was considered as one of the most important measurement properties (Terwee et al., 2007), was rated as positive in more than half (59%) of the included tools. Although only 24% of the tools (Boele et al., 2019; Girgis et al., 2011; Hodgkinson et al., 2007; Yan, 2019b) had a positive rating in construct validity based on the criterion of more than 75% variance contribution rate, 50% variance contribution rate was also recommended by a Chinese expert (Minglong, 2010), which means more included tools would have the positive rating. However, the shortage is that some psychometric properties were less assessed in the included tools, such as the criterion validity, reproducibility (reliability and agreement), floor and ceiling effects, interpretability and responsiveness. Previous study (Terwee et al., 2007) has shown that discriminative questionnaires require a high level of reliability, and evaluative questionnaires require a level of agreement as well as responsiveness. Therefore, these above warrants researcher further attention and development in the future.

### 5.3 Needs themes of the needs assessment tools

In our study, 27 needs themes extracted from the included tools were matched to six levels of Maslow's 7 hierarchy of needs, besides aesthetic needs. This means that the needs assessment scope of these tools is comparatively comprehensive. Especially the most frequent four needs themes (psychological‐emotional, information, communication and healthcare professionals support) we extracted from the included tools were consistent with the unmet needs reported in the previous studies (Akgun‐Citak et al., 2020; Hodson et al., 2019; Lee & Lee, 2020; Sarabia‐Cobo et al., 2020; Wang et al., 2018). However, whether the aesthetic needs assessment was ignored by the previous studies or it was not cared about by informal caregivers needs further exploration. Besides, the self‐realization needs level was found to have the largest number of needs themes, then followed by the level of love and belonging, which is different from previous reports, such as the knowledge need of patient’ symptoms management and medicine administration (Akgun‐Citak et al., 2020). This implies that the development of need assessment tools might facilitate caregivers or assessors to recognize the new unmet needs. What's more, according to Family System Theory (Keith, 1980), the needs satisfaction of both caregivers and patients must be taken into account in the same time in order to maintain a good care relationship. Thus, researches about informal caregivers' needs assessment and intervention in the context of good relationship setting are suggested.

### 5.4 Limitations

We limited studies in English and Chinese, which might have missed some relevant tools in other languages. Another limitation is that we did not contact the study authors to identify the missing information on the tool's psychometric properties.

## 6 CONCLUSION AND IMPLICATIONS

In our systematic review, a total of 17 tools from six countries were developed to assess informal caregivers' needs of CNCDs patients. Twenty‐seven needs themes were extracted and were matched to six levels of Maslow's hierarchy of needs theory, besides aesthetics needs. Although the common psychometric properties, such as content validity, internal consistency and construct validity were considered in most tools, the overall quality of the included tools was not too satisfactory because of lacking of high percentage of positive rating indicators and other psychometric properties, such as reproducibility and responsiveness. All of the above might be helpful to the needs assessment tools choice of clinical nurse and provide further improvement prospects for tool developments, such as the comprehensiveness of needs assessment and the quality of psychometric properties.

## AUTHOR CONTRIBUTIONS

YPP and FYY carried out contributions to the conception of the work. YPP, MMZ, GQY, DXB and FYY were involved in acquisition, analysis, interpretation of data for the work, drafting the manuscript or revising it critically for important intellectual content, final approval of the version to be published and agreement to be accountable for all aspects of the work in ensuring that questions related to the accuracy or integrity of any part of the work are appropriately investigated and resolved.

## FUNDING INFORMATION

2018 the Postgraduate Tutor guidance ability improvement fund project of Shandong Province, China. Funding provided by Department of Education of Shandong Province. Grant number SDYY18171. 2021 graduate science and technology innovation project (School of Nursing, Binzhou Medical University). Grant number HYCX2021‐007.

## CONFLICT OF INTEREST STATEMENT

The authors declare no conflict of interest.

## ETHICS STATEMENT

None.

## Supporting information


Appendix S1.

File S1.

## Data Availability

The data that supports the findings of this study are available in the supplementary material of this article.
