# Supplementary material for: Assessing the needs of informal caregivers of patients with chronic non‐communicable diseases: A systematic review of self‐assessment tools
Source: Nurs Open. 2023 Oct 3;10(12):7467–86. doi: 10.1002/nop2.2008 (PMC10643841; doi:10.1002/nop2.2008)
Supplement: Supplementary file 1 — Appendix S1. [file NOP2-10-7467-s001.docx]

**Appendix S1**

**Retrieval Strategy**

| **A: Search history from PubMed August 2021**  #1 Non communicable diseases [MeSH Terms] OR noncommunicable disease [MeSH Terms]  #2 Non communicable diseases [MeSH Terms] OR noncommunicable disease [MeSH Terms]  #3 #1 OR #2  #4 caregivers [MeSH Terms]  #5 family members [Title/Abstract] OR Spouses [Title/Abstract] OR family caregivers [Title/Abstract] OR caregivers [Title/Abstract]  #6 #4 OR #5  #7 needs assessment [MeSH Terms]  #8 professional needs [Title/Abstract] OR unmet needs [Title/Abstract] OR comprehensive needs[Title/Abstract] OR care needs [Title/Abstract] OR needs assessment [Title/Abstract]  #9 #7 OR #8  #10 instrument [Title/Abstract] OR tool [Title/Abstract] OR scale [Title/Abstract]  #11 #6 AND #9 AND #10  #12 #3 AND #6 AND #9 AND #10  #13 #11 + #12 |
| --- |
| **B: Search history from Embase August 2021**  #1 'Non communicable diseases':ab,ti OR 'noncommunicable disease':ab,ti  #2 'caregivers'/exp  #3 'spouses':ab,ti OR 'family caregivers':ab,ti OR 'family members':ab,ti OR 'caregivers':ab,ti  #4 #2 OR #3  #5 'needs assessment'/exp  #6 'care needs':ab,ti OR 'needs assessment':ab,ti OR 'professional needs':ab,ti OR 'comprehensive needs':ab,ti OR 'unmet needs':ab,ti  #7 #5 OR #6  #8 'scale':ab,ti OR 'tool':ab,ti OR 'instrument':ab,ti  #9 #4 AND #7 AND #8  #10 #1 AND #4 AND #7 AND #8  #11 #9 + #10  **C: Search history from CINAHL August 2021**  #1 AB Non communicable diseases OR AB noncommunicable disease  #2 TI Non communicable diseases OR TI noncommunicable disease  #3 #1 OR #2  #4 AB caregivers OR AB spouses OR AB family caregivers OR AB family members  #5 TI caregivers OR TI spouses OR TI family caregivers OR TI family members  #6 #4 OR #5  #7 AB needs assessment OR AB unmet needs OR AB comprehensive needs OR AB professional needs OR AB care needs  #8 TI needs assessment OR TI professional needs OR TI unmet needs OR TI comprehensive needs OR TI care needs  #9 #7 OR #8  #10 AB scale OR AB tool OR AB instrument  #11 TI scale OR TI tool OR TI instrument  #12 #10 OR #11  #13 #6 AND #9 AND #12  #14 #3 AND #6 AND #9 AND #12  #15 #13 + #14  **D: Search history from Web of Science August 2021**  #1 AB= (Non communicable diseases OR noncommunicable disease)  #2 TI= (Non communicable diseases OR noncommunicable disease)  #3 #1 OR #2  #4 AB= (caregivers OR spouses OR family caregivers OR family members)  #5 TI= (caregivers OR spouses OR family caregivers OR family members)  #6 #4 OR #5  #7 AB=(needs assessment OR unmet needs OR comprehensive needs OR professional needs OR care needs)  #8 TI=(needs assessment OR unmet needs OR comprehensive needs OR professional needs OR care needs)  #9 #7 OR #8  #10 AB=( scale OR tool OR instrument)  #11 TI=( scale OR tool OR instrument)  #12 #10 OR #11  #13 #6 AND #9 AND #12  #14 #3 AND #6 AND #9 AND #12  #15 #13 + #14  **E: Search history from Wan Fang Data August 2021**  #1 主题:("慢性非传染性疾病") OR 主题:("慢性疾病") OR 主题:("慢性病")  #2 主题:("照顾者") OR 题名或关键词:("照护者") OR 题名或关键词:("家属") OR 题名或关键词:("家庭照护者") OR 题名或关键词:("配偶")  #3 主题:("需求评估") OR 题名或关键词:("综合需求") OR 题名或关键词:("未满足需求") OR 题名或关键词:("照护需求") OR 题名或关键词:("专业需求")  #4 题名或关键词:("量表") OR 题名或关键词:("问卷") OR 题名或关键词:("工具")  #5 #2 AND #3 AND #4  #6 #1 AND #2 AND #3 AND #4  #7 #5 + #6  **F: Search history from CNKI August 2021**  #1 SU% = '慢性非传染性疾病' OR TKA = '慢性非传染性疾病' OR SU% = '慢性疾病' OR TKA = '慢性疾病' OR SU% = '慢性病' OR TKA = '慢性病'  #2 SU% = '照顾者' OR TKA = '照顾者' OR SU% = '照护者' OR TKA = '照护者' OR SU% = '家属' OR TKA = '家属' OR SU% = '家庭照护者' OR TKA = '家庭照护者' OR SU% = '配偶' OR TKA = '配偶'  #3 SU%= '需求评估' OR TKA = '需求评估' OR SU% = '综合需求' OR TKA = '综合需求' OR SU% = '未满足需求' OR TKA = '未满足需求' OR SU% = '照护需求' OR TKA = '照护需求' OR SU% = '专业需求' OR TKA = '专业需求'  #4 KY = '量表' OR KY = '问卷' OR KY= '工具'  #5 #2 AND #3 AND #4  #6 #1 AND #2 AND #3 AND #4  #7 #5 + #6  **G: Search history from CQVIP August 2021**  #1 R=(慢性非传染性疾病 OR 慢性疾病 OR 慢性病)  #2 M=(慢性非传染性疾病 OR 慢性疾病 OR 慢性病)  #3 #1 OR #2  #4 R=(照顾者 OR 照护者 OR 家属 OR 家庭照护者 OR 配偶)  #5 M=(照顾者 OR 照护者 OR 家属 OR 家庭照护者 OR 配偶)  #6 #4 OR #5  #7 R=(需求评估 OR 综合需求 OR 未满足需求 OR 照护需求 OR 专业需求)  #8 M=(需求评估 OR 综合需求 OR 未满足需求 OR 照护需求 OR 专业需求)  #9 #7 OR #8  #10 R=(量表 OR 问卷 OR 工具)  #11 M=(量表 OR 问卷 OR 工具)  #12 #10 OR #11  #13 #6 AND #9 AND #12  #14 #3 AND #6 AND #9 AND #12  #15 #13 + #14  **H: Search history from CBM August 2021**  #1 "慢性非传染性疾病"[常用字段:智能] OR "慢性疾病"[常用字段:智能] OR "慢性病"[常用字段:智能]  #2 "照顾者"[关键词] OR "照护者"[常用字段:智能] OR "家属"[常用字段:智能] OR "家庭照护者"[常用字段:智能] OR "配偶"[常用字段:智能]  #3 "需求评估"[关键词] OR "综合需求"[常用字段:智能] OR "未满足需求"[常用字段:智能] OR "照护需求"[常用字段:智能] OR "专业需求"[常用字段:智能]  #4 "量表"[常用字段:智能] OR "问卷"[常用字段:智能] OR "工具"[常用字段:智能]  #5 #2 AND #3 AND #4  #6 #1 AND #2 AND #3 AND #4  #7 #5 + #6 |
